# Exogenous Application of Growth Enhancers Mitigate Water Stress in Wheat by Antioxidant Elevation

**DOI:** 10.3389/fpls.2016.00597

**Published:** 2016-05-04

**Authors:** Hamid Nawaz, Azra Yasmeen, Muhammad A. Anjum, Nazim Hussain

**Affiliations:** ^1^Department of Agronomy, Bahauddin Zakariya UniversityMultan, Pakistan; ^2^Department of Horticulture, Bahauddin Zakariya UniversityMultan, Pakistan

**Keywords:** enzymes, non-enzymes, benefit cost ratio, growth regulators, irrigation water-regimes

## Abstract

The present study was conducted to investigate the response of two wheat cultivars (AARI-11 and Millat-11) to a foliar application of four growth enhancers which include: {H_2_O (water), MLE30 (moringa leaf extract), KCl (potassium chloride), and BAP (benzyl-amino purine)}, within the six irrigation water-regimes which are applied at the various critical growth stages such as crown root initiation (CRI), tillering (T), booting (B), and heading (H). Irrigation water-regimes include: CRI+T+B, CRI+T, CRI+B, T+B, T+H, and control (CRI+T+B+H). The growth enhancers i.e., H_2_O, MLE30 (1:30), KCl (2%), and BAP (50 mg L^−1^) were applied @ 500 L ha^−1^ at tillering and heading stages. The results demonstrated some increased quantities of both enzymatic (superoxide dismutase, peroxidase, catalase) and non-enzymatic (ascorbic acid, phenol) antioxidants in leaves of AARI-11 when MLE30 was applied under T+B and T+H irrigation water-regimes. Similar results were also observed in the case of leaf chlorophyll “*a*” and “*b*” and K^+^ contents in both cultivars under control, T+B and CRI+T+B irrigation water regimes. AARI-11 produced the highest biological and grain yield, due to the application of MLE30 and BAP under control, CRI+T+B, T+B, and T+H irrigation water-regimes. However, KCl lagged behind among the treatments set for both cultivars under all the irrigation water-regimes. Foliar spray of MLE30 remained prominent growth enhancer and stresses mitigating agent under water deficit conditions particularly under T+B and T+H irrigation water-regimes. Moreover, economic analysis indicated that the foliar application of MLE30 is a cost effective and environment friendly strategy for the maximum yield and income.

## Introduction

Wheat (*Triticum aestivum* L.) is one of the most important feeding cereals for one-fifth of the world population (FAO, 2011). Therefore, wheat crops require a special attention for an incremental production in order to ease the food security issues for the world population which is growing so rapidly. Today, Wheat plants are facing oxidative damage at cellular level which reduces leaf surface area, crop growth rate, net assimilation rate, leaf chlorophyll and nutrient contents in grains due to insufficient availability of water (Araus et al., 2003). For mitigating oxidative stress, application of irrigations at vegetative and reproductive growth stages is a sensitive tool for obtaining optimum yields. Irrigation water-regimes at the critical growth stages of wheat minimize severe losses of grain yield as at pre-anthesis (1–30%) and post-anthesis (58–92%) stages (Farooq et al., 2014). Hence, to determine the most critical growth stages of wheat for irrigation management and utilization of available water resources is necessary to maximize crop yield.

The behavior of plants in surviving and producing good yield under limited water stress is called drought tolerance (Turner, 1979). Plants' tolerance against environmental stresses can be increased by the exogenous application of certain growth enhancers like proline, amino acids, ABA, glycine betaine, BAP, silicon, soluble sugars, humic acid, and potassium (Farooq et al., 2009). Appropriate concentrations of these synthetic enhancers could promote the growth of plants and ameliorate water deficit stress by interfering in metabolic and photosynthesis processes through osmotic adjustment, scavenging ROS, increasing enzymatic and non-enzymatic antioxidants and proteins (Bohnert and Jensen, 1996). Exogenous foliar spray of growth enhancers at the critical growth stages of wheat (tillering, booting, heading, milking), is one of the most significant parts which increase antioxidants status against reactive oxygen species under water deficit condition (Yasmeen et al., 2012). Whereas, using these synthetic growth enhancers like BAP or KCl may cause various drastic effects on wheat grain quality, the consumers' health and benefit cost ratio. However, the applications of naturally occurring growth enhancers like moringa leaf extract (MLE) can be environment friendly and economically feasible. Moringa (*Moringa oleifera*) is a well-known native tree of southern Punjab (Pakistan) and has proved as an excellent growth enhancer containing K, Ca, Fe, amino acids, ascorbates, and growth regulating hormones such as zeatin (Fuglie, 2000). Yasmeen et al. (2012) screened out the positive impact of MLE at various critical growth stages of wheat under saline stress in control conditions (laboratory) and suggested the best application time at tillering and heading. The responses of MLE application to ameliorate drought impacts in wheat at critical growth stages have not been well established yet. Therefore, the present study was conducted to evaluate the performance of MLE as an organically natural plant growth enhancer in comparison with BAP and KCl for improving the productivity of wheat under various irrigation water-regimes at the critical growth stages.

## Materials and methods

**Figure 1:**
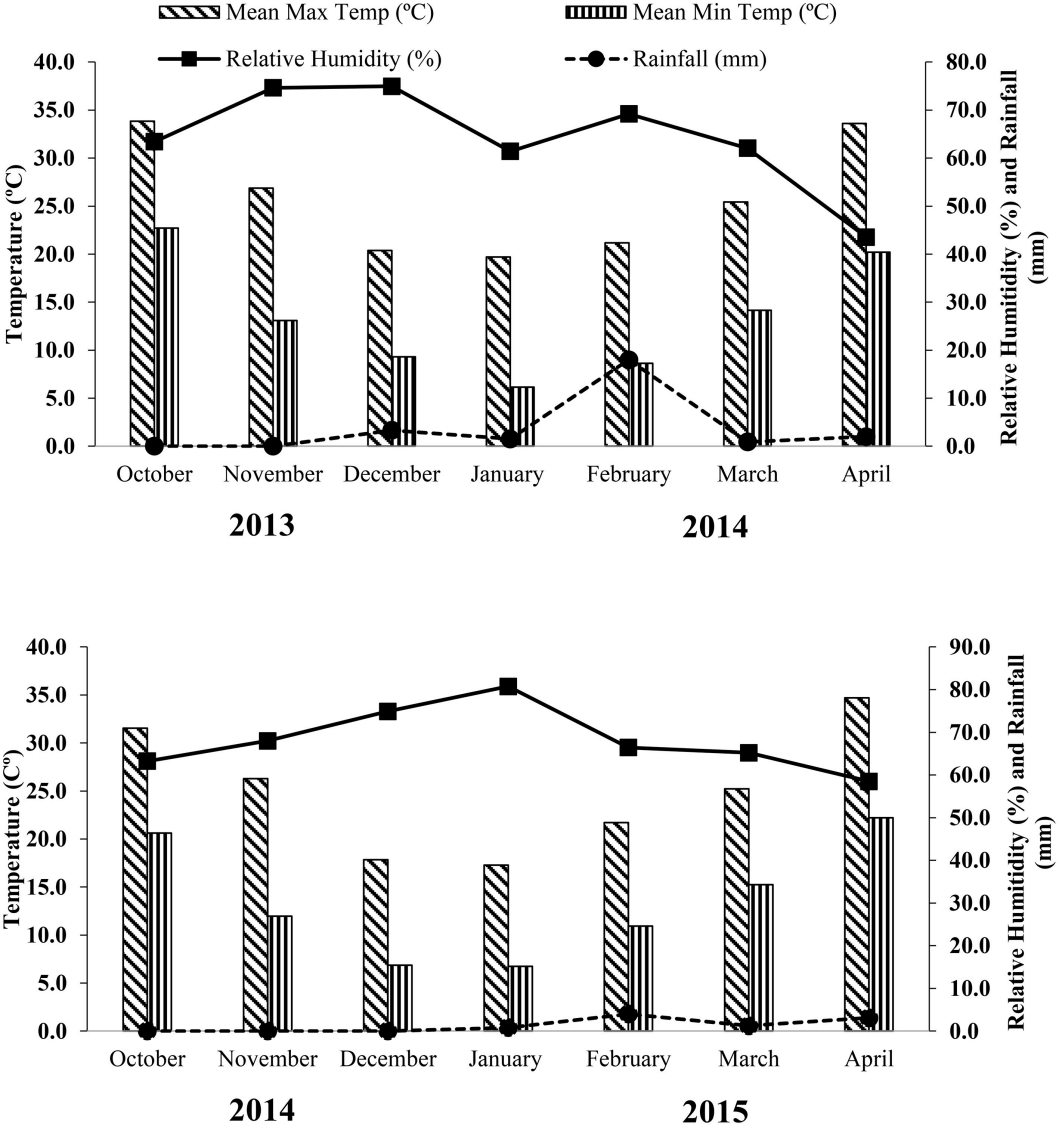
**Meteorological data for growing period of wheat crop during the years 2013-2014 and 2014-2015**.

The experiments were conducted at the Agronomic Experimental Area, Bahauddin Zakariya University Multan (71.43°E, 30.2°N and 122 m above sea level), Pakistan, during the winter seasons of 2013–2014 and 2014–2015. The region is located in semi-arid and sub-tropical climate and data of mean annual temperature. An average rainfall and relative humanity, during the both years of crop growing period is presented in Figure 1. The soil belongs to Sindhlianwali soil series (fine silty, mixed, hyperthermic, sodic haplocambids) in USDA Hap-lic Yermosols in FAO classification. It was characterized after analyzing the samples taken from different locations of the experimental site. Soil was clay loam having EC_*e*_ 2.42 dS m^−1^ and pH 8.7, organic matter 0.83–0.88%, total nitrogen 0.05–0.06%, available phosphorus 5.50–5.54 mg kg^−1^, available potassium 300–303 mg kg^−1^ and zinc 0.36–0.39 mg kg^−1^ during both years of trials. The trial comprised two wheat cultivars AARI-11 (drought tolerant) and Millat-11 (drought sensitive; Nawaz et al., 2015). Six irrigation water-regimes were adopted based on the critical growth stages of wheat {Crown Root Initiation (CRI), Tillering (T), Booting (B), and Heading (H) stages} i.e., irrigations applied at CRI+T+B+H (control), irrigations applied at CRI+T+B, irrigations applied at CRI+B, irrigations applied at CRI+H, irrigations applied at T+B and irrigations applied at T+H. The Foliar Application of four growth enhancers i.e. H_2_O (control), MLE30 (1:30), KCl (2%), BAP (50 mg L^−1^) @ 500 L ha^−1^ were applied using garden sprayer (Flat Fan Nozzle). The response of wheat plants to the application of growth enhancers under drought stress or the effect of the growth enhancers on the planted wheat was recorded by measuring growth, yield and biochemical parameters in both the cultivars.

The procedure described by Yasmeen et al. (2012) for the preparation of moringa leaf extract, was followed. Fresh young leaves were collected from moringa trees grown in the Experimental Area of Faculty of Agricultural Sciences and Technology, Bahauddin Zakariya University Multan, Pakistan and stored in a freezer at −80°C. The leaves were crushed through locally available juicer machine; the extract was purified by centrifuging at 8000 rpm for 20 min and diluted 30 times by adding distilled water. Foliar spray of growth enhancers H_2_O, MLE30, KCl, and BAP was were applied at the tillering and heading stages of the wheat cultivars.

Pre-soaking irrigation of 10 cm depth was applied on the soil to prepare the favorable seedbed conditions for the experiment. After observing the soil at a workable moisture level, it was plowed twice, followed by planting. The seed rate was 125 kg ha^−1^ and NPK applied at 120-100-62.5 kg ha^−1^ by using fertilizers urea, single super phosphate and potassium sulfate, respectively. Whole recommended phosphorus, potash and one-third of nitrogen were applied as basal dose and the remaining nitrogen was applied in two splits during the growing period of wheat crop. During both years of trial, slight rains in the growing period occurred but its intensity was not enough to change the soil moisture level under applied water deficit stress condition at the critical growth stages. Intercultural practices and crop protection measures were practiced as per requirement of the crop uniformly for all experimental plots.

Flag leave samples were collected randomly in the morning time (temp. 20 ± 2°C) and stored in polythene bags at −80°C for antioxidants analysis after just 1 week of the last irrigation and foliar application of growth enhancers at the heading stage. The protocol devised by Bradford (1976) was applied to quantify the total soluble proteins (TSP). For determination of endogenous enzymatic and non-enzymatic antioxidants, standard protocols were adopted to measure peroxidase (POD), catalase (CAT) (Chance and Maehly, 1955), superoxide dismutase (SOD) (Giannopolitis and Reis, 1997), ascorbic acid (AsA) (Ainsworth and Gillespie, 2007), and total phenolic contents (TPC) (Waterhouse, 2001). Leaf chlorophyll (“*a*” and “*b*”) (Nagata and Yamashita, 1992) and potassium (K^+^) contents (Rashid, 1986) were determined as per given standard procedures. The number of the productive tillers (m^−2^) and the number of grains per spike and 1000-grain weight were also recorded. The mature crop was harvested on the 1st and 2nd fortnight of April during the first and second years of the trail respectively, and threshed manually to determine grain yield, biological yield, and harvest index.

The total expenditures of the wheat production were calculated including land rent, seedbed preparation, seed cost, sowing labor, fertilizers, irrigations, weeds preventive measures and harvesting charges of the crops for economic analysis. Gross income was calculated using recent market prices of wheat grains and straw. Net income was obtained by subtracting the total expenditures from gross income and benefit cost-ratio estimated at a ratio of gross income and total expenditures. Data were computed and analyzed statistically using Fisher's analysis of variance technique and LSD test (*p* < 0.05) to compare differences among the mean values (Steel et al., 1997). Moreover, Microsoft Excel Program 2013 was used for the graphical presentation of meteorological data.

## Results

### Antioxidants activities

Application of the growth stimulators significantly enhanced the TSP during imposed water deficit stressed levels. However, MLE30 and BAP sprays were more effective in this regard during the both years of study. The maximum TSP was obtained from the foliar application of MLE30 in Millat-11 under irrigation water-regimes of T+B and T+H (Table 1). The effect of exogenously applied stimulators on antioxidants status was found statistically significant (Tables 1, 2). The contents of enzymatic and non-enzymatic antioxidants were increased with the imposition of water deficit stress at the critical growth stages in both wheat cultivars. Among the treatments, MLE30 and BAP application revealed maximum enzymatic activities i.e. SOD, POD, and CAT under irrigated water-regimes of T+B, T+H, and CRI+T respectively, in AARI-11 as compared to Millat-11 during the both years (Tables 1, 2). Irrigation at T+B stages showed a dominant and gradual rise in AsA content in flag leaf of wheat. The maximum contents of AsA (non-enzymatic antioxidants) were observed due to foliar application of MLE30 under all irrigation water-regimes in both cultivars during the both years of trial (Table 2). However, increased content of TPC was obtained under applied irrigations at T+B and T+H stages from applied treatment of MLE30, followed by BAP and KCl in AARI-11 during the both years (Table 2).

**Table 1:** **Influence of different foliar agents on total soluble protein (TSP), superoxide Dismutase (SOD) and peroxidase (POD) of wheat cultivars under various applied irrigation water-regimes during 2013-2014 (Year-I) and 2014-2015 (Year-II)**.

			**Year-I**									**Year-II**								
	**Foliar application**	**H**_**2**_**O**_**2**_		**MLE**		**KCl**		**BAP**			**H**_**2**_**O**_**2**_		**MLE30**		**KCl**		**BAP**		
	**Wheat cultivars**	**AARI-11**	**Millat-11**	**AARI-11**	**Millat-11**	**AARI-11**	**Millat-11**	**AARI-11**	**Millat-11**	**Mean**	**AARI-11**	**Millat-11**	**AARI-11**	**Millat-11**	**AARI-11**	**Millat-11**	**AARI-11**	**Millat-11**	**Mean**
TSP (mg g^−1^) Irrigation water-regimes	Control (CRI+T+B+H)	1.20o.q	0.97w	1.34d.h	1.19o.q	1.25k.n	1.20n.q	1.30g.k	1.10uv	1.19d	1.95d.k	1.70r	2.02c.f	1.87k.n	1.98c.h	1.85l.o	1.97c.j	1.79n.q	1.89bc
	CRI+T+B	1.31g.k	1.13r.u	1.35c.g	1.16p.s	1.30g.k	1.09uv	1.27i.l	1.10uv	1.21cd	1.91h.m	1.75p.r	2.02c.e	1.83m.p	1.97c.i	1.76o.r	1.94e.l	1.77o.r	1.87cd
	CRI+B	1.27i.l	1.13r.u	1.38cd	1.24l.o	1.36c.f	1.16q.t	1.32f.j	1.10t.v	1.24b	1.80n.q	1.72qr	2.02c.e	1.73qr	1.99c.h	1.88j.n	1.99c.h	1.91h.m	1.88b.d
	CRI+H	1.31f.j	1.10uv	1.34d.h	1.20n.q	1.31g.k	1.18p.r	1.30h.k	1.12s.u	1.23bc	1.83m.p	1.58s	2.02c.f	1.87k.n	1.92g.l	1.87k.n	1.98c.h	1.77o.r	1.85d
	T+B	1.40bc	1.21m.p	1.63a	1.37c.e	1.30g.k	1.26j.m	1.44b	1.34d.h	1.37a	2.05bc	1.87k.n	2.30a	2.04b.d	1.97c.j	1.93f.l	2.12b	2.01c.g	2.03a
	T+H	1.27i.l	1.06v	1.35c.g	1.05v	1.32e.i	1.21m.q	1.31f.j	1.24l.o	1.23bc	1.88i.n	1.75p.r	2.05bc	1.91h.m	2.03b.d	1.83m.p	1.99c.h	1.77o.r	1.90b
	Mean	1.29c	1.10f	1.40a	1.20d	1.31bc	1.18de	1.32b	1.17e		1.90c	1.73e	2.07a	1.87cd	1.98b	1.85d	1.99b	1.84d	
	Mean	1.20c		1.30a		1.24b		1.25b			1.81c		1.97a		1.91b		1.92b		
		LSD 0.05*p* = W^*^F^*^V 0.0557, W 0.0197, F 0.0161, F^*^V 0.0227									LSD 0.05*p* = W^*^F^*^V 0.0908, W 0.0321, F 0.0262, F^*^V 0.0371								
SOD (IU min^−1^mg^−1^ protein) Irrigation water-regimes	Control (CRI+T+B+H)	55.25h	29.52m	75.34e	66.68f	62.39f	46.96i	62.89f	56.45gh	56.93d	66.25r.t	40.52x	86.34j.l	77.68mn	73.39n.q	57.96uv	73.89n.p	67.45rs	67.93e
	CRI+T+B	44.62i	30.56m	74.71e	74.39e	64.18f	56.54gh	67.08f	64.98f	59.63c	69.32p.r	59.15u	86.27j.l	101.55cd	66.82r.t	61.70tu	75.61no	84.82j.l	75.65d
	CRI+B	46.32i	36.15kl	63.27f	78.55e	43.82ij	38.70jk	52.61h	61.82fg	52.65e	62.62s.u	48.56w	92.7f.i	92.39g.i	82.18lm	74.54n.p	85.08j.l	82.98k.m	77.63c
	CRI+H	42.53ij	31.23lm	86.97d	75.10e	47.11i	30.29m	66.10f	73.87e	56.65d	62.53s.u	51.23w	106.9b	95.10e.g	67.11rs	50.29w	86.10j.l	93.87e.h	76.65cd
	T+B	99.75b	52.89h	125.59a	85.10d	94.15c	46.65i	104.34b	74.70e	85.39a	106.75bc	59.89u	132.59a	92.10g.i	101.15d	53.65vw	111.34b	81.70lm	92.39a
	T+H	63.90f	44.24i	74.53e	87.17d	64.53f	46.67i	73.81e	64.21f	64.88b	87.90i.k	68.24qr	98.53de	111.17b	88.53h.j	70.67o.r	97.81d.f	88.21i.k	88.88b
	Mean	58.72f	37.43h	83.40a	77.832 b	62.69e	44.30g	71.13c	66.00d		75.89f	54.60h	100.57a	95.00b	79.86e	61.47g	88.30c	83.17d	
	Mean	48.07d		80.61a		53.50c		68.57b			65.24d		97.78a		70.66c		85.73b		
		LSD 0.05*p* = W^*^F^*^V5.3867, W 1.9045, F 1.5550, F^*^V 2.1991									LSD 0.05*p* = W^*^F^*^V 5.0811, W 1.86001, F 1.4690, F^*^V 2.20470								
POD (mmol min^−1^ mg protein^−1^) Irrigation water-regimes	Control (CRI+T+B+H)	22.65h.k	17.02z.b	27.67b	22.30j.l	24.26fg	21.52l.o	25.55cd	20.25q.s	22.65b	28.26lm	24.25u	31.67de	26.30p.s	26.65n.q	25.52st	29.55h.k	21.02 w	26.65d
	CRI+T+B	21.51l.o	18.01w.y	24.51ef	19.84r.t	21.34m.p	17.268y.a	22.91h.j	18.05w.y	20.43d	26.34p.s	23.05v	29.51h.k	24.84tu	26.51o.r	22.26v	27.91lm	23.01v	25.43e
	CRI+B	20.06rs	17.44x.a	22.46i.l	20.52p.r	21.18n.q	17.93w.z	20.66o.r	17.82x.z	19.76e	26.32p.s	24.34u	29.36i.k	27.42m.o	27.56mn	24.72tu	28.08lm	24.83tu	26.58d
	CRI+H	22.18j.m	18.37v.x	24.11fg	21.58l.o	22.65h.k	19.48s.u	22.92h.j	18.88u.w	21.27c	30.45f.h	26.17q.s	31.91d	29.38i.k	29.98g.i	26.68n.q	30.72e.g	27.28m.p	29.07b
	T+B	25.28de	20.69o.r	31.58a	24.23fg	23.52gh	21.69k.n	26.27c	23.41g.i	24.59a	31.52de	29.69h.j	39.58a	32.23d	33.28c	28.69kl	34.27b	31.41d.f	32.59a
	T+H	19.05t.v	14.34c	22.41j.l	16.26b	19.93r.t	16.64ab	19.84r.t	17.46x.a	18.24f	28.83j.l	23.24v	31.31d.f	25.16tu	27.95lm	25.54r.t	28.74j.l	26.36p.s	27.14c
	Mean	21.79c	17.65f	25.46a	20.79d	22.15c	19.09e	23.03b	19.31e		28.62c	25.12f	32.22a	27.56d	28.65c	25.57e	29.88b	25.65e	
	Mean	19.72d		19.72d		19.72d		19.72d			26.87c		29.89a		27.11c		27.77b		
		LSD 0.05*p* = W^*^F^*^V 0.9619, W 0.3401, F 0.2777, F^*^V 0.3927									LSD 0.05*p* = W^*^F^*^V 0.9875, W 0.3491, F 0.2851, F^*^V 0.4032								

Means not sharing the same letters in a group differ significantly at 5% probability level. W, Irrigation Water-regimes; F, Foliar agents; V, Wheat cultivars.

**Table 2:** **Influence of different foliar agents on catalase (CAT), ascorbic acid (AsA) and total phenolic contents (TPC) of wheat cultivars under various applied irrigation water-regimes during 2013-2014 (Year-I) and 2014-2015 (Year-II)**.

			**Year-I**									**Year-II**								
	**Foliar application**	**H**_**2**_**O**_**2**_		**MLE**		**KCl**		**BAP**			**H**_**2**_**O**_**2**_		**MLE30**		**KCl**		**BAP**		
	**Wheat cultivars**	**AARI-11**	**Millat-11**	**AARI-11**	**Millat-11**	**AARI-11**	**Millat-11**	**AARI-11**	**Millat-11**	**Mean**	**AARI-11**	**Millat-11**	**AARI-11**	**Millat-11**	**AARI-11**	**Millat-11**	**AARI-11**	**Millat-11**	**Mean**
CAT (μ mol min^−1^ mg protein^−1^) Irrigation water-regimes	Control (CRI+T+B+H)	8.20u	9.11r.t	10.89q	9.55	8.75s.u	8.91r.u	9.42rs	8.30u	9.14f	16.60lm	9.03w	18.42k	10.31uv	15.18no	8.85wx	16.62lm	9.55vw	13.07d
	CRI+T+B	13.60no	8.66tu	14.64m	9.24r.t	13.51no	8.26u	13.70n	8.54tu	11.27e	10.95tu	6.88yz	13.99pq	7.80xy	11.63st	6.15za	12.42rs	5.77a	9.45e
	CRI+B	16.80l	14.09mn	20.25h	16.72l	19.28ij	14.57m	18.67jk	14.73m	16.89d	19.15k	12.34rs	22.79hi	14.82n.p	21.79ij	12.78r	21.09j	13.03qr	17.22c
	CRI+H	21.61g	17.12l	23.69f	19.92hi	22.05g	18.65jk	22.07g	18.04k	20.39b	24.79ef	13.12qr	26.80d	14.94n.p	25.69e	14.89n.p	25.66e	15.38no	20.16b
	T+B	27.17c	21.36g	33.69a	24.81e	26.61cd	21.52g	29.68b	23.88f	26.09a	30.40c	21.05j	37.44a	24.47fg	29.60c	21.20j	33.00b	23.54gh	27.59a
	T+H	24.00f	10.98q	25.96d	12.14p	24.87e	12.45p	24.84e	12.87op	18.51c	23.72f.h	14.34op	25.85de	16.88l	24.15fg	15.67mn	24.16fg	15.21no	20.00b
	Mean	18.56d	13.55h	21.52a	15.40e	19.18c	14.06g	19.73b	14.39f		20.94c	12.79g	24.21a	14.87d	21.34c	13.26f	22.16b	13.75e	
	Mean	16.06d		18.46a		16.62c		17.06b			16.86d		19.54a		17.30c		17.95b		
		LSD 0.05*p* = W^*^F^*^V 0.7493, W 0.2649, F 0.2163, F^*^V 0.3059									LSD 0.05*p* = W^*^F^*^V 1.0834, W 0.3830, F 0.3128, F^*^V 0.4423								
AsA (m. mole g^−1^) Irrigation water-regimes	Control (CRI+T+B+H)	31.56t	34.20s	55.76p	38.96r	31.76t	34.26s	38.26r	38.56r	37.92f	37.36z	40.00y	48.46v	45.66w	39.66y	42.16x	38.80yz	38.53yz	41.33e
	CRI+T+B	65.40ij	55.76p	69.46f	57.40n	65.56ij	55.90op	72.26b.d	57.10no	62.35e	71.20m.o	61.56rs	76.16f.h	64.10q	73.46i.k	63.80q	72.80j.m	57.66t	67.59d
	CRI+B	71.33c.e	58.70m	71.60b.e	65.20j	71.26de	58.90lm	71.46c.e	60.10l	66.07b	71.56mn	64.70q	79.26cd	69.80o	75.10g.i	70.23no	71.86k.n	63.30qr	70.72b
	CRI+H	67.56gh	49.90q	71.60b.e	67.83g	69.70f	50.10q	71.40c.e	66.56hi	64.33d	73.36i.l	55.70u	78.30c.e	74.53h.j	77.60d.f	58.00t	71.93k.n	67.06p	69.56c
	T+B	76.33a	70.50ef	77.06a	72.80b	76.26a	71.20de	76.46a	71.16de	73.97a	82.13b	76.30fg	83.76ab	79.50c	84.16a	79.10cd	77.00ef	71.66l.n	79.20a
	T+H	65.76ij	58.90lm	72.56bc	63.10k	67.20gh	62.33k	71.33c.e	62.76k	65.49c	77.13ef	64.50q	78.30c.e	71.90k.n	79.16cd	66.80p	72.00k.m	60.66s	71.30b
	Mean	62.99d	54.66h	69.67a	60.88e	63.62c	55.45g	66.86b	59.37f		68.79c	60.46f	74.04a	67.58d	71.52b	63.35e	67.40d	59.81f	
	Mean	58.82d		65.28a		59.53c		63.12b			64.62c		70.81a		67.43b		63.60d		
		LSD 0.05*p* = W^*^F^*^V 1.2371, W 0.4374, F 0.3571, F^*^V 0.5050									LSD 0.05*p* = W^*^F^*^V 1.7452, W 0.6170, F 0.5038, F^*^V 0.7125								
TPC (mg g^−1^) Irrigation water-regimes	Control (CRI+T+B+H)	1.80st	1.29uv	2.91l.n	2.43o.q	1.87rs	1.40u	2.73m.o	2.15qr	2.07d	2.58k.p	1.47qr	3.02h.n	2.32l.q	2.01n.r	1.29r	2.84i.n	2.04n.r	2.20c
	CRI+T+B	1.50tu	1.01v	3.30i.k	2.70m.o	1.47tu	0.98v	2.98k.m	2.35pq	2.04d	1.80o.r	1.22r	3.48e.k	2.73j.o	1.65p.r	1.19r	2.78j.o	2.35l.q	2.15c
	CRI+B	3.30i.k	2.56op	4.07de	3.67fg	3.43g.i	2.98k.m	3.79ef	3.31h.k	3.39c	3.12h.l	3.55d.k	4.29c.g	3.83d.i	3.10h.m	3.97c.h	3.32f.l	4.30b.f	3.68b
	CRI+H	4.40cd	3.64f.h	4.45bc	3.61f.i	3.63f.i	2.57n.p	3.36g.j	3.47f.i	3.64b	3.66d.j	3.10h.m	4.54a.d	3.65d.j	3.13h.l	2.41l.q	3.45e.k	3.51d.k	3.43b
	T+B	3.61f.i	3.03j.m	4.89a	4.42c	3.68fg	3.08j.l	4.80a	4.22cd	3.97a	4.27c.g	3.26g.l	5.46a	3.84d.i	4.34b.f	3.31f.l	5.33ab	4.45a.e	4.28a
	T+H	4.43c	2.91l.n	4.78ab	3.44g.i	3.47f.i	1.96rs	4.84a	3.31h.k	3.64b	3.71d.j	3.04h.n	4.89 a.c	2.87i.n	3.58d.k	2.09m.r	4.95a.c	2.97h.n	3.51b
	Mean	3.17d	2.41f	4.06a	3.38c	2.93e	2.16g	3.75b	3.14d		3.19c	2.61de	4.28a	3.21c	2.97cd	2.38e	3.78b	3.27c	
	Mean	2.79c		3.72a		2.54d		3.44b			2.90b		3.74a		2.67b		3.53a		
		LSD 0.05*p* = W^*^F^*^V 0.3364, W 0.1189, F 0.0971, F^*^V 0.1373									LSD 0.05*p* = W^*^F^*^V 1.0308, W 0.3644, F 0.2976, F^*^V 0.4208								

Means not sharing the same letters in a group differ significantly at 5% probability level. W, Irrigation Water-regimes; F, Foliar agents; V, Wheat cultivars.

### Leaf chlorophyll contents

Chlorophyll contents “*a*” and “*b*” were found significantly higher, due to foliar application of MLE30 and BAP under applied irrigation regimes of control, T+ B and T+H stages in AARI-11 during the both years of study (Table 3). The results also illustrated that the chlorophyll contents “*a*” and “*b*” decreased in Millat-11 under all irrigation water-regimes but due to the foliar application of MLE30 and BAP, their levels were maintained (Table 3).

**Table 3:** **Influence of different foliar agents on chlorophyll “*a* & *b*” and K^+^ contents of wheat cultivars under various applied irrigation water-regimes during 2013-2014 (Year-I) and 2014-2015 (Year-II)**.

			**Year-I**									**Year-II**								
	**Foliar application**	**H**_**2**_**O**_**2**_		**MLE**		**KCl**		**BAP**			**H**_**2**_**O**_**2**_		**MLE30**		**KCl**		**BAP**		
	**Wheat cultivars**	**AARI-11**	**Millat-11**	**AARI-11**	**Millat-11**	**AARI-11**	**Millat-11**	**AARI-11**	**Millat-11**	**Mean**	**AARI-11**	**Millat-11**	**AARI-11**	**Millat-11**	**AARI-11**	**Millat-11**	**AARI-11**	**Millat-11**	**Mean**
Chlorophyll “*a*” (mg g^−1^) Irrigation water-regimes	Control (CRI+T+B+H)	1.07m.q	0.85q.s	2.53a	2.15cd	2.20b.d	0.80q.s	2.33a.c	1.07m.q	1.62a	2.53a.c	1.13n.r	2.63ab	2.03de	1.76e.h	1.73e.i	2.27cd	1.66g.j	1.97a
	CRI+T+B	1.07m.q	0.83q.s	1.57g.k	1.91d.f	2.13cd	0.89q.s	2.12cd	0.87q.s	1.42bc	1.57g.l	0.95q.t	1.68f.j	1.66g.j	1.18m.r	0.88r.t	1.42i.n	0.93r.t	1.28d
	CRI+B	0.91p.r	1.00n.q	1.47h.l	1.46h.l	1.19l.p	1.55g.k	1.39i.l	1.69e.i	1.33cd	1.27l.q	1.56g.l	1.56g.l	1.48h.m	0.99o.s	1.02o.s	1.48h.m	1.70f.j	1.38cd
	CRI+H	0.39t	1.20l.p	0.61st	2.45ab	0.87q.s	1.29k.n	1.21l.o	1.63f.j	1.21e	0.97p.s	1.48h.m	0.72s.u	2.64ab	0.49u	1.39j.n	1.32k.o	1.82e.g	1.35cd
	T+B	1.21l.o	1.38j.l	2.08cd	1.35j.m	1.97de	0.79q.s	1.72e.h	1.31k.m	1.48b	2.41bc	0.91r.t	2.74a	2.26cd	1.29l.p	0.97p.s	2.54a.c	1.19m.r	1.79b
	T+H	0.40t	0.98o.q	2.35a.c	1.44h.l	1.42i.l	0.64r.t	1.78e.g	0.89q.s	1.24de	1.64g.k	0.75s.u	2.57a.c	1.55g.l	0.62tu	1.10n.r	2.00d.f	1.00o.s	1.40c
	Mean	0.84e	1.04d	1.77a	1.79a	1.63b	0.99d	1.76a	1.24c		1.73c	1.13e	1.98a	1.93ab	1.05e	1.18e	1.84bc	1.38d	
	Mean	0.94d		1.78a		1.31c		1.50b			1.43c		1.96a		1.12d		1.61b		
		LSD 0.05*p* = W^*^F^*^V 0.2950, W 0.1043, F 0.0852, F^*^V 0.1204									LSD 0.05*p* = W^*^F^*^V 0.3309, W 0.1170, F 0.0955, F^*^V 0.1351								
Chlorophyll “*b*” (mg g^−1^) Irrigation water-regimes	Control (CRI+T+B+H)	0.21q	0.86c	0.61u	0.89b	0.59x	0.72k	0.49e	0.70n	0.63a	1.03b	0.47st	1.06a	0.87f	0.89e	0.85fg	0.87f	0.75i	0.85a
	CRI+T+B	0.60w	0.52d	0.72j	0.70m	0.52c	0.61t	0.48g	0.56z	0.59c	0.72j	0.61no	0.84g	0.79h	0.64m	0.70k	0.60n.p	0.65lm	0.69b
	CRI+B	0.37l	0.57y	0.73i	0.65q	0.72l	0.30n	0.74f	0.32m	0.55d	0.55q	0.64lm	0.91d	0.72j	0.90de	0.37v	0.76i	0.39v	0.65c
	CRI+H	0.46h	0.20r	0.80e	0.60v	0.61v	0.45i	0.65p	0.44j	0.53e	0.47st	0.21y	0.62n	0.61no	0.51r	0.46tu	0.59op	0.45u	0.49e
	T+B	0.53b	0.25p	0.90a	0.68o	0.82d	0.54a	0.74h	0.44k	0.61b	0.30w	0.55q	0.95c	0.84g	0.59p	0.70k	0.49s	0.76i	0.63d
	T+H	0.27o	0.09s	0.74g	0.64s	0.72j	0.49f	0.65r	0.59x	0.52f	0.28x	0.11z	0.75i	0.66l	0.73j	0.31w	0.66lm	0.21y	0.46f
	Mean	0.41h	0.41g	0.75a	0.69b	0.66c	0.52e	0.62d	0.51f		0.56e	0.43g	0.85a	0.75b	0.71c	0.56e	0.66d	0.53f	
	Mean	0.41d		0.72a		0.59b		0.57c			0.49d		0.80a		0.64b		0.60c		
		LSD 0.05*p* = W^*^F^*^V 0.173, W 0.164, F 0.422, F^*^V 0.0153									LSD 0.05*p* = W^*^F^*^V 0.0193, W 0.1112, F 0.232, F^*^V 0.1209								
K^+^ contents (mg g^−1^) Irrigation water-regimes	Control (CRI+T+B+H)	2.00d	1.90e	2.33a	2.20b	2.10c	1.80f	2.20b	2.10c	2.07a	2.43c.g	2.37c.i	3.01a	2.59b.d	2.46c.g	2.24e.l	2.51c.e	2.44c.g	2.50a
	CRI+T+B	1.80f	1.10m	2.20b	1.40j	1.60h	1.30k	1.70g	1.40j	1.56c	2.24e.l	2.14g.o	2.61b.d	2.39c.h	2.21e.n	2.14f.o	2.64bc	2.33c.j	2.34b
	CRI+B	0.60q	0.60q	1.10m	1.00n	0.80p	0.80p	0.90o	0.90o	0.83f	1.57t.v	1.50uv	1.72q.v	1.59s.v	1.48uv	1.39v	1.58s.v	1.50uv	1.54f
	CRI+H	1.10m	1.10m	1.20l	1.40j	1.00n	1.10m	1.10m	1.10m	1.13e	1.70r.v	1.70r.v	1.89m.t	1.99j.r	1.66r.v	1.84o.t	1.70r.v	1.78p.u	1.78e
	T+B	1.60h	1.50i	2.20b	1.80f	1.70g	1.60h	2.00d	1.70g	1.76b	2.48c.f	1.76p.u	2.87ab	2.07h.p	2.28d.k	1.97k.r	2.22e.m	2.05i.q	2.21c
	T+H	1.30k	1.20l	1.40j	1.30k	1.20l	1.10m	1.30k	1.20l	1.25d	1.95k.r	1.81o.u	2.08h.p	1.91l.s	1.87n.t	1.71q.v	1.96k.r	1.96k.r	1.91d
	Mean	1.40c	1.23e	1.73a	1.51b	1.40c	1.28d	1.53b	1.40c		2.06b	1.88c	2.36a	2.09b	1.99bc	1.88c	2.10b	2.01bc	
	Mean	1.31c		1.62a		1.34c		1.46b			1.97bc		2.23a		1.94c		2.05b		
		LSD 0.05*p* = W^*^F^*^V 0.0969, W 0.0343, F 0.0280, F^*^V 0.0396									LSD 0.05*p* = W^*^F^*^V 0.3404, W 0.1204, F 0.0983, F^*^V 0.1390								

Means not sharing the same letters in a group differ significantly at 5% probability level. W, Irrigation Water-regimes; F, Foliar agents; V, Wheat cultivars.

### Leaf K^+^ content

The MLE30 application increased leaf K^+^ content under imposed irrigation water-regimes, but the maximum leaf K^+^ content was observed in AARI-11 plants under the applied irrigation water-regimes of control, CRI+T+B and T+B respectively, in the both years. The least K^+^ contents were observed under the irrigation water-regimes applied at CRI+T, CRI+B, and CRI+H stages in both cultivars during the both years of trial (Table 3).

### Yield and yield components

Foliar applications, irrigation water-regimes and wheat cultivars significantly affected the number of fertile tillers (Table 4). Results demonstrated that the application of MLE30 and BAP with irrigation water-regimes of CRI+T+B+H, CRI+T+B, and T+B resulted in the maximum fertile tillers in AARI-11 during the both growing years. The minimum number of fertile tillers was observed when irrigations were applied at CRI+B and T+H stages in both cultivars during the year-I but fertile tillers increased by the application of growth stimulators during the year-II, even though the irrigations were applied at CRI+B and T+H stages (Table 4). Results revealed that the number of grains per spike with the irrigation water-regimes at control, CRI+T+B and T+B stages were non-significant in both cultivars. Among the foliar sprays, performance of MLE30 was better than BAP, KCl, and H_2_O and produced the maximum number of grains per spike in both cultivars under all irrigation water-regimes (Table 4). Foliar growth agents under the applied irrigations at CRI+T+B+H, T+B, T+H critical growth stages in the studied wheat cultivars significantly affected yield components (Table 5). The results depicted that 1000-grain weight in AARI-11 was the maximum under the applied irrigation water-regimes of control, followed by T+B and T+H especially by MLE30 treatment during the both years of study. Yield parameters i.e. grain yield, biological yield and harvest index clearly demonstrated significant results among the wheat cultivars grown under various irrigation water-regimes (Table 5). Among the growth stimulators, MLE30 and BAP applied to AARI-11, resulted in the maximum grain yield and harvest index when the irrigations were applied at CRI+T+B+H, CRI+T+B, and T+B stages during the both years. Harvest index of wheat cultivars during the both years of trial at the applied irrigation water-regimes of control and T+B was the maximum due to the application of MLE30 (Table 5).

**Table 4:** **Influence of different foliar agents on fertile tillers (m^−2^), grain Spike^−1^ and 1000 grain weight (g) of wheat cultivars under applied irrigation water-regimes during 2013-2014 (Year-I) and 2014-2015 (Year-II)**.

			**Year-I**									**Year-II**								
	**Foliar application**	**H**_**2**_**O**_**2**_		**MLE**		**KCl**		**BAP**			**H**_**2**_**O**_**2**_		**MLE30**		**KCl**		**BAP**		
	**Wheat cultivars**	**AARI-11**	**Millat-11**	**AARI-11**	**Millat-11**	**AARI-11**	**Millat-11**	**AARI-11**	**Millat-11**	**Mean**	**AARI-11**	**Millat-11**	**AARI-11**	**Millat-11**	**AARI-11**	**Millat-11**	**AARI-11**	**Millat-11**	**Mean**
Fertile tillers (m^−2^) Irrigation water-regimes	Control (CRI+T+B+H)	300f.h	342a.c	348ab	351a	309e.g	336a.c	343a.c	330b.d	332a	270g.j	332a.e	352a	355a	290f.i	323a.f	335a.d	336a.d	324a
	CRI+T+B	330b.d	312d.f	342a.c	333a.c	334a.c	297f.h	342a.c	308e.g	325b	312c.f	305d.g	331a.e	337a.d	337a.d	268ij	348ab	314b.f	319a
	CRI+B	289gh	290gh	302f.h	287h	295f.h	301f.h	295f.h	292gh	294c	324a.f	241j.l	344a.c	242j.l	322a.f	232k.m	329a.e	253jk	286b
	CRI+H	202j.l	209j	240i	196j.l	201j.l	201j.l	200j.l	197j.l	206d	204m.p	211l.o	224k.n	200m.p	204m.p	199m.p	206m.p	203m.p	206c
	T+B	339a.c	239i	340a.c	238i	329b.d	248i	323c.e	247i	288c	291f.i	292f.i	306d.f	291f.i	298e.i	304d.h	270h.j	297e.i	293b
	T+H	188kl	184l	205jk	188kl	191j.l	187kl	188kl	184l	189e	190n.q	186o.q	209l.p	193n.q	193n.q	174pq	194n.q	159q	187d
	Mean	275b	263c	296a	265c	276b	261c	282b	259c		265c	261cd	294a	269bc	274bc	250d	280ab	260cd	
	Mean	269b		281a		269b		271b			263b		282a		262b		270b		
		LSD 0.05*p* = W^*^F^*^V 20.179, W 7.1343, F 5.8251, F^*^V 8.2379									LSD 0.05*p* = W^*^F^*^V 35.170, W 12.434, F 10.153, F^*^V 14.358								
Grain spike^−1^ Irrigation water-regimes	Control (CRI+T+B+H)	36v	37t	50a	45d	41k	37t	46c	40n	41b	42kl	43jk	55a	51bc	47fg	43jk	47f.h	45g.i	46b
	CRI+T+B	42g	36v	47b	45d	44e	37t	46c	40n	42a	48ef	41lm	52b	50cd	49de	42kl	51bc	45hi	47a
	CRI+B	34z	39o	46c	42hi	38qr	42hi	42hi	39o	40c	44ij	39no	48ef	48ef	45hi	41lm	46gh	43jk	44d
	CRI+H	31b	39p	42g	41k	36w	40m	42i	41l	39d	37p	44ij	48ef	47fg	41lm	46gh	47fg	46gh	44d
	T+B	38q	34a	42f	42h	40n	35x	41k	38r	39e	39no	44ij	51bc	47fg	43jk	47fg	47fg	44ij	45c
	T+H	37u	37s	42f	39o	35y	37t	42j	38p	38f	38op	41l.n	47fg	41lm	39no	40m.o	46gh	41k.m	41e
	Mean	36h	37g	45a	42c	39e	38f	43b	39d		41e	42e	50a	47b	44c	43d	47b	44c	
	Mean	37d		44a		38c		41b			42d		49a		43c		46b		
		LSD 0.05*p* = W^*^F^*^V 0.1351, W 0.0478, F 0.0390, F^*^V 0.0552									LSD 0.05*p* = W^*^F^*^V 1.7024, W 0.6019, F 0.4914, F^*^V 0.6950								
1000-Grain weight (g) Irrigation water-regimes	Control (CRI+T+B+H)	37.30l	37.86j	51.60a	43.20c	40.40f	37.50k	43.00d	39.70g	41.32a	39.16j	39.76h	48.76a	41.06d	41.66c	38.76k	44.36b	40.36g	41.74a
	CRI+T+B	33.06v	35.66p	43.76b	37.16m	35.56q	35.06r	35.96o	36.26n	36.56b	29.86I	30.66F	34.46u	36.56q	33.86x	29.26J	30.56G	35.40s	32.58c
	CRI+B	31.36b	30.46d	32.76x	34.36s	29.76f	30.76c	31.46a	32.46z	31.67d	33.26Z	32.36B	32.80A	33.80y	31.00E	32.00C	29.90I	31.50D	32.07d
	CRI+H	23.80o	24.80m	30.16e	32.90w	26.26k	24.80m	27.16j	26.30k	27.02f	25.66q	26.66O	28.50K	30.06H	27.50M	26.06P	27.30N	27.66L	27.42f
	T+B	28.00i	28.76g	33.40u	39.46h	32.60y	28.06h	33.10v	34.06t	32.18c	34.96t	37.56n	40.90e	37.66M	36.80p	36.30r	37.36o	34.30w	36.98b
	T+H	32.46z	23.66p	42.46e	24.96l	37.16m	22.16q	39.36i	24.26n	30.81e	34.36v	25.56r	40.70f	25.66Q	38.36l	23.40S	39.56i	22.10T	31.21e
	Mean	31.00f	30.20g	39.02a	35.34b	33.62d	29.72h	35.01c	32.17e		32.88e	32.10f	37.68a	34.13d	34.86b	30.96h	34.84c	31.88g	
	Mean	30.60d		37.18a		31.67c		33.59b			32.49d		35.91a		32.91c		33.36b		
		LSD 0.05*p* = W^*^F^*^V 0.0446, W 0.0158, F 0.0129, F^*^V 0.0182									LSD 0.05*p* = W^*^F^*^V 0.0452, W 0.0160, F 0.0131, F^*^V 0.0185								

Means not sharing the same letters in a group differ significantly at 5% probability level. W, Irrigation Water-regimes; F, Foliar agents; V, Wheat cultivars.

**Table 5:** **Influence of different Foliar agents on grain yield (t/ha), biological yield (t/ha) and harvest index (%) of wheat cultivars under applied irrigation water-regimes during 2013-2014 (Year-I) and 2014-2015 (Year-II)**.

			**Year-I**									**Year-II**								
	**Foliar application**	**H**_**2**_**O**_**2**_		**MLE**		**KCl**		**BAP**			**H**_**2**_**O**_**2**_		**MLE30**		**KCl**		**BAP**		
	**Wheat cultivars**	**AARI-11**	**Millat-11**	**AARI-11**	**Millat-11**	**AARI-11**	**Millat-11**	**AARI-11**	**Millat-11**	**Mean**	**AARI-11**	**Millat-11**	**AARI-11**	**Millat-11**	**AARI-11**	**Millat-11**	**AARI-11**	**Millat-11**	**Mean**
Grain yield (t/ha) Irrigation water-regimes	Control (CRI+T+B+H)	4.55h.l	4.07n.p	5.65a	4.41j.n	4.86e.h	4.29k.o	4.92d.g	4.31j.o	4.63a	4.88f.k	4.25p.r	5.84a	5.40bc	4.88f.k	4.38n.r	5.37b.d	5.28b.e	5.04a
	CRI+T+B	4.38j.n	2.08v	5.47ab	4.62g.k	5.22b.d	3.14tu	5.28bc	4.18m.p	4.29c	4.55k.q	5.18c.f	5.19c.f	4.41m.r	4.56k.q	4.90f.k	5.00e.i	5.37b.d	4.89b
	CRI+B	4.48j.m	2.20v	4.97c.f	3.93pq	4.50i.m	2.94u	4.58g.k	3.88p.r	3.93e	4.68h.n	4.61j.p	5.40bc	5.11c.g	4.63i.o	3.42u	4.95e.j	4.08rs	4.61c
	CRI+H	4.40j.n	3.14tu	5.17b.e	4.62f.k	3.41st	4.20m.p	4.65f.j	4.57g.k	4.27c	4.51l.q	4.41m.r	4.76g.m	4.85f.l	4.41m.r	3.67tu	4.58k.q	4.58k.q	4.47d
	T+B	4.59g.k	3.43st	5.25b.d	4.44j.m	5.09c.e	3.55rs	5.10c.e	4.32j.o	4.47b	4.68h.n	4.11rs	5.63ab	5.40bc	4.41m.r	4.38n.r	5.12c.g	5.37b.d	4.89b
	T+H	4.01o.q	3.69q.s	4.83e.i	4.46j.m	4.21l.p	3.44st	4.34j.o	4.01o.q	4.12d	4.21qr	4.27o.r	5.03d.h	5.17c.f	3.79st	4.78g.l	5.06c.g	4.60j.p	4.61c
	Mean	4.40d	3.10g	5.22a	4.41cd	4.55c	3.59f	4.81b	4.21e	4.59d	4.47d	5.31a	5.06b	4.45d	4.26e	5.01bc	4.88c	4.59d	
	Mean	3.75d		4.82a		4.07c		4.51b			4.53c		5.18a		4.35d		4.94b		
		LSD 0.05*p* = W^*^F^*^V 0.3470, W 0.1227, F 0.1002, F^*^V 0.1416									LSD 0.05*p* = W^*^F^*^V 0.3659, W 0.1294, F 0.1056, F^*^V 0.1494								
Biological yield (t/ha) Irrigation water-regimes	Control (CRI+T+B+H)	12.03f	9.93s	13.33a	11.33i	12.83d	9.33x	12.96c	10.66n	11.55a	13.42b.d	11.69g.m	14.47a	12.14f.i	13.30b.e	11.81g.k	13.83ab	10.84l.r	12.69a
	CRI+T+B	11.16j	7.96E	11.66g	10.03r	11.13j	8.53C	11.36i	9.16z	10.12d	12.60d.g	10.19q.v	13.58a.c	11.77g.l	12.77c.f	11.82g.k	12.80c.f	11.41h.o	12.12b
	CRI+B	10.20q	8.86A	11.66g	11.13j	11.60h	9.13z	11.16j	9.73u	10.43c	9.91s.v	8.27w	12.80c.f	10.93k.r	10.79m.s	9.80t.v	11.17j.p	10.40p.u	10.51e
	CRI+H	9.80t	7.63G	10.16q	10.06r	9.83t	7.83F	9.90s	8.76B	9.25f	11.51h.m	10.10r.v	13.40b.d	11.43h.n	11.36i.o	10.48o.t	10.56n.t	11.16j.p	11.25cd
	T+B	10.73m	8.03D	13.03b	11.16j	11.03k	9.13z	12.46e	9.63v	10.65b	11.07j.q	10.19q.v	13.81ab	11.89f.j	12.11f.i	10.54n.t	12.21f.i	10.79m.s	11.57c
	T+H	10.33p	7.63G	10.96l	9.46w	10.43o	8.86A	10.73m	9.23y	9.70e	10.90k.r	9.47uv	13.54bc	12.33f.h	11.36i.o	9.45v	12.45e.g	10.15q.v	11.21d
	Mean	10.71d	8.34h	11.80a	10.53e	11.14c	8.80g	11.43b	9.53f		11.57c	9.98e	13.60a	11.75c	11.95bc	10.65d	12.17b	10.79d	
	Mean	9.52d		11.16a		9.97c		10.48b			10.78c		12.67a		11.30b		11.48b		
		LSD 0.05*p* = W^*^F^*^V 0.0500, W 0.0177, F 0.0144, F^*^V 0.0204									LSD 0.05*p* = W^*^F^*^V 0.9298, W 0.3287, F 0.2684, F^*^V 0.3796								
Harvest index (%) Irrigation water-regimes	Control (CRI+T+B+H)	37.11f.m	40.51a.g	41.93a	41.78ab	33.17n.s	40.62a.f	41.00a.e	37.84c.l	39.24a	41.55a.e	33.67n.p	45.11ab	40.06c.j	42.02a.d	33.75n.p	41.86a.d	37.41f.n	39.43a
	CRI+T+B	34.69l.r	39.05a.j	38.08b.l	31.90p.s	34.86k.q	39.75a.h	37.46d.m	41.22a.d	37.13b	38.44d.m	31.14op	44.08a.c	40.50c.i	30.15pq	40.13c.j	38.65d.m	40.99c.f	38.01ab
	CRI+B	39.89a.h	32.87o.s	41.53a.c	35.53j.p	35.81i.o	27.77t	39.62a.i	31.58q.t	35.57c	34.17n.p	34.80m.o	39.05d.l	39.78d.k	36.54h.n	36.32j.n	35.52l.n	36.34j.n	36.56b
	CRI+H	37.71d.m	29.72st	38.58a.k	34.42l.r	36.78g.n	30.92r.t	37.90c.l	33.24n.s	34.91c	34.79m.o	20.47r	41.09b.f	39.29d.l	39.29d.l	26.60q	40.61c.h	36.68g.n	34.85c
	T+B	35.64j.p	37.23e.m	39.64a.h	38.06b.l	36.29h.o	36.15h.o	37.54d.m	36.40h.o	37.12b	40.78c.g	26.89q	45.20a	36.07j.n	41.92a.d	30.16pq	41.00b.f	37.38f.n	37.42b
	T+H	36.98f.n	32.67o.s	40.25a.g	37.59d.m	34.81k.q	33.93m.r	38.02b.l	35.17k.q	36.18bc	36.81g.n	34.82m.o	39.18d.l	36.20j.n	37.43f.n	36.45i.n	35.68k.n	37.51f.n	36.76b
	Mean	37.00b	35.34cd	40.00a	36.55bc	35.28cd	34.86d	38.59a	35.91b.d		37.76b	30.30d	42.28a	38.65b	37.89b	33.90c	38.89b	37.71b	
	Mean	36.17bc		38.27a		35.07c		37.25ab			34.03d		40.47a		35.90c		38.30b		
		LSD 0.05*p* = W^*^F^*^V 3.8185, W 1.3500, F 1.1023, F^*^V 1.5589									LSD 0.05*p* = W^*^F^*^V 4.1122, W 1.4539, F 1.1871, F^*^V 1.6788								

Means not sharing the same letters in a group differ significantly at 5% probability level. W, Irrigation Water-regimes; F, Foliar agents; V, Wheat cultivars.

### Benefit cost ratio

Economic analysis showed that the MLE30 foliar application was comparatively the most cost effective technology to obtain the maximum benefit cost ratio (BCR) with irrigation water-regimes of CRI+T and T+B after control (Table 6).

**Table 6:** **Economic analysis (average of both cultivars) for the impact of foliar spray under various irrigation water-regimes at the critical growth stages of wheat**.

**Treatment**		**Total expenditure (US$ ha**^**−1**^**)**		**Gross income (US$ ha**^**−1**^**)**		**Net income (US$ ha**^**−1**^**)**		**Benefit cost ratio**	
		**2013-14**	**2014-15**	**2013-14**	**2014-15**	**2013-14**	**2014-15**	**2013-14**	**2014-15**
H_2_O foliar spray	Control	629.27	629.27	1282.33	1046.57	653.06	417.29	2.04	1.66
	CRI+T+B	615.64	615.64	1129.78	1124.85	514.14	509.21	1.84	1.83
	CRI+B	602.00	602.00	989.42	928.70	387.42	326.70	1.64	1.54
	CRI+H	602.00	602.00	891.71	1074.46	289.71	472.46	1.48	1.78
	T+B	602.00	602.00	886.56	1075.68	284.56	473.68	1.47	1.79
	T+H	602.00	602.00	1033.09	1038.68	431.09	436.68	1.72	1.73
MLE30 foliar spray	Control	633.55	606.55	997.95	1367.99	391.40	761.45	1.65	2.26
	CRI+T+B	620.18	620.18	1183.97	1282.92	563.78	662.74	1.91	2.07
	CRI+B	606.55	606.55	995.79	1006.53	389.24	399.98	1.64	1.66
	CRI+H	606.82	633.82	1479.73	1311.78	845.92	677.96	2.33	2.07
	T+B	606.55	606.55	1172.01	1266.43	565.46	659.88	1.93	2.09
	T+H	606.55	606.55	1019.57	1249.79	413.02	643.25	1.68	2.06
KCl foliar spray	Control	642.91	642.91	1219.56	1160.59	576.65	517.68	1.90	1.81
	CRI+T+B	629.27	629.27	969.19	1160.91	339.92	531.64	1.54	1.84
	CRI+B	615.64	615.64	1048.31	903.26	432.67	287.62	1.70	1.47
	CRI+H	615.64	615.64	731.44	1036.67	115.81	421.03	1.19	1.68
	T+B	615.64	615.64	793.32	1038.09	177.68	422.45	1.29	1.69
	T+H	615.64	615.64	930.64	1171.35	315.00	555.72	1.51	1.90
BAP foliar spray	Control	670.18	670.18	1445.62	1018.51	775.44	348.33	2.16	1.52
	CRI+T+B	656.55	656.55	1130.94	1297.51	474.40	640.96	1.72	1.98
	CRI+B	642.91	642.91	1025.59	940.16	382.68	297.25	1.60	1.46
	CRI+H	642.91	642.91	892.51	981.87	249.61	338.96	1.39	1.53
	T+B	642.91	642.91	977.00	1253.91	334.09	611.00	1.52	1.95
	T+H	642.91	642.91	1041.61	1300.17	398.70	657.26	1.62	2.02

## Discussion

Oxidative stress under water deficit conditions is characterized as an imbalance between production of ROS and quenching activity of antioxidant system which possesses a serious threat to plant cellular membranes and other cellular organelles like proteins, DNA, etc. (White et al., 1993). The ability of plant to scavenge toxic/active ionic forms of oxygen radicles has seemed to be an important consideration of its tolerance to environmental stresses including water deficit stress. Plant antioxidants defense system including enzymatic (SOD, CAT, POD) and non-enzymatic (AsA, TPC) ones has been proved a possible protective measure against oxidative damages by inhibiting release of ROS (Mittler, 2002). The present study demonstrated that increased contents of antioxidants (enzymatic and non-enzymatic) by foliar application of growth enhancers especially MLE30 and BAP at various sensitive growth stages of wheat cultivars under water deficit conditions, is the most effective strategy. Exogenous applications of MLE30, BAP, KCl and H_2_O to AARI-11 plants helped in ameliorating the water stress effects during the water deficit conditions and motivated the enzymatic antioxidants defense system to maintain its ionic homeostasis status. The maximum release of SOD, POD, CAT enzymatic contents due to the application of MLE30 and BAP probably has the ability to mitigate the effect of water stress during the applied irrigation water-regimes at T+B and T+H stages of AARI-11 plants (Farooq et al., 2009). On the other hand, non-enzymatic antioxidants (AsA) played crucial role in scavenging ROS and triggering the antioxidant defensive system against water deficit stress in wheat plants. The foliar application with MLE30 and BAP enhanced the production of AsA which increased the plant tolerance and improved photosynthetic activity in AARI-11 cultivar under irrigation water-regimes at T+B and T+H stages (Hanaa et al., 2008). The foliar application of MLE30 played an important role in protective measures against oxidative damage stress in wheat cultivar AARI-11 and enhanced the activities of TPC under irrigation water-regimes of T+B and T+H during the both years. The ability of MLE30 foliar agent to initiate the positive release of antioxidants is due to the presence of zeatin in it, which reduced the water stress and also promoted leaf chlorophyll contents “*a*” and “*b*” that has a vital role in photosynthesis process (Foidle et al., 2001).

Crop yield is based on chlorophyll contents and photosynthesis rate during the normal environmental conditions. The present study described that increased chlorophyll contents “*a*” and “*b*” in AARI-11 plants were due to their larger leaf area, and exogenous application of growth enhancer facilitated the photosynthetic rate under water deficit conditions especially at irrigation water-regimes of T+B after control and CRI+T+B stages, respectively (Ali et al., 2011). Similarly, Yasmeen et al. (2013) reported significance of MLE30 as moringa leaves contain a heavy amount of mineral contents including K^+^ which is an excellent plant growth enhancer/regulator. Leaf K^+^ content in AARI-11 plants with the application of MLE30 and BAP attributed direct increase in the K^+^ content which ultimately enhanced the uptake of K^+^ during the stomatal conductance (Cakmak, 2005).

Wheat yield depends on numbers of fertile/productive tillers and grain weight at harvest. The maximum production of fertile tiller in AARI-11 was due to its greater tolerance against water deficit stress and in addition to foliar application, of MLE30 and BAP at tillering and heading stages which resulted in increased number of grains per spike and 1000-grain weight under control, followed by T+H and T+B irrigation water-regimes (Baque et al., 2006). Biological yield was significantly prominent in AARI-11 plants. It might be possible that the foliar application of MLE30 and BAP at heading stage mitigated the water scarcity impact in wheat which increased grain yield not only in control, but also in T+B and T+H irrigation water-regimes (Yasmeen et al., 2012). Tillering and heading are the most important stages for the foliar application of growth regulators. At the tillering stage, MLE30 promoted the booting and similarly at the heading stages enhanced the grain filling and milking which led to increased biological yield and grain yield (Farooq et al., 2014). The foliar application also increased the harvest index (HI) during the both years, but MLE30 growth stimulator gave the maximum HI possibly due to its role in dry matter accumulation under various irrigation water-regimes i.e. control, CRI+T+B, T+B, and T+H, respectively, (Madani et al., 2010). Economic analysis illustrated that foliar application of MLE30 was a cost effective strategy for an increased benefit cost ratio of wheat.

## Conclusion

The foliar application of naturally occurring MLE30 growth enhancer at the tillering and heading stages of wheat cv. AARI-11 ameliorated the water deficit stress by enhancing the antioxidants contents to protect it against oxidative damage which modulated yield related components under irrigation water-regimes at T+B and T+H over control (CRI+T+B+H).

## Future research needs

Mechanism of photosynthetic product distribution and assimilation needs to be investigated after MLE application.

## Author contributions

NH Supervisor Helped in supervised the research project and guide in conducting the research in the Field. HN student, performed all the activities during the research including research planning, lab analysis, write up. AY Co-supervisor, helped in lab analysis and data analysis. MA Help in review the article and guide in improving the article.

### Conflict of interest statement

The authors declare that the research was conducted in the absence of any commercial or financial relationships that could be construed as a potential conflict of interest.

## Abbreviations

AsA: ascorbic acid
BAP: benzyl-amino purine
B: booting
BCR: benefit cost ratio
CRI: crown root initiation
Chl.: chlorophyll
CAT: catalase
H: heading
MLE: moringa leaf extract
POD: peroxidase
ROS: reactive oxygen species
SOD: superoxide dismutase
T: tillering
TPC: total phenolic contents
TSP: total soluble proteins.
